# Screening Strains of the Mulberry Silkworm, *Bombyx mori*, for Thermotolerance

**DOI:** 10.1673/031.011.11601

**Published:** 2011-09-12

**Authors:** Savarapu Sugnana Kumari, Sure Venkata Subbarao, Sunil Misra, Upadyayula Suryanarayana Murty

**Affiliations:** Biology Division, Indian Institute of Chemical Technology, Tarnaka, Hyderabad - 500 007, Andhra Pradesh, India

**Keywords:** genetic traits, rearing performance, survival rate, temperature stress, thermotolerant

## Abstract

A tropical climate prevails in most of the sericultural areas in India, where temperature increases during the summer lead to adverse effects on temperate bivoltine silkworm rearing and cause crop losses. Screening for thermotolerance in the silkworm, *Bombyxmori* L. (Lepidoptera: Bombycidae) is an essential prerequisite for the development of thermotolerant breeds/hybrids. In the current study, the aim was to identify potential bivoltine silkworm strains specific for tolerance to high temperature. The third day of fifth stage silkworm larvae of bivoltine strains were subjected to high temperature of 36 ± 1° C with RH of 50 ± 5 % for six hours (10:00–16:00) every day until spinning for three consecutive generations. Highly significant differences were found among all genetic traits of bivoltine silkworm strains in the treated groups. Three groups of silkworm resulted including susceptible, moderately tolerant, and tolerant by utilizing pupation rate or survival rate with thermal stress as the index for thermotolerance. Furthermore, based on the overall silkworm rearing performance of nine quantitative genetic traits such as larval weight, cocoon yield by number and weight, pupation, single cocoon and shell weight, shell ratio, filament length and denier, three bivoltine silkworm strains, BD2-S, SOF-BR and BO_2_ were developed as having the potential for thermotolerance. The data from the present study enhance knowledge for the development of thermo tolerant silkworm breeds/ hybrids and their effective commercial utilization in the sericulture industry.

## Introduction

The mulberry silkworm, *Bombyx mori* L. (Lepidoptera: Bombycidae), is a very heat-sensitive organism. Intensive and careful domestication over centuries has apparently deprived this commercial insect of the opportunity to acquire thermotolerance. This vulnerability is more pronounced in bivoltine strains compared to polyvoltine strains. Thus, among many factors responsible for poor performance of the bivoltine strains under tropical conditions, the major one is lack of thermotolerance. Many quantitative characters decline sharply at higher temperatures. Therefore, one of the key considerations in developing bivoltine hybrids for tropics could be the need for thermotolerant bivoltine strains. The recent advances in silkworm breeding and those in stress-induced protein synthesis have opened up new avenues to evolve robust productive silkworm hybrids (Evgenev et al. 1987; Kumar et al. 1989; Coulon-Bublex and Mathelin 1991; Wu and Hou 1993; Joy and Gopinathan 1995; Kumar et al. 2001, 2002, 2003; Reddy et al. 2002; Vasudha et al. 2006; Srivastava et al. 2007; Moghaddam et al. 2008).

Sericulture in India is practiced predominantly in tropical environmental regions such as Karnataka, Tamil Nadu, Andhra Pradesh, and West Bengal and to a limited extent in the temperate environment of Jammu and Kashmir. This situation provides scope for creating polyvoltine × bivoltine hybrids as a commercial venture as hybrids are hardy and have the ability to survive and reproduce under varied or fluctuating environmental climatic conditions.

However, hybrid quality is low when compared to the existing international standards. For example, cross breeds of polyvoltine female × bivoltine male are generally reared in these regions during the summer, but the quality of cocoon production is not as high as it is for bivoltine silkworm hybrids (Ramesha et al. 2009). Bivoltine silkworm breeds are known for their qualitative and quantitative traits in the sericulture industry. During the last decade, a number of silkworm hybrids have been developed (Basavaraja et al. 1995; Data et al. 1997) and selected for exploitation at the field level during favorable seasons. These productive bivoltine breeds are susceptible to varied environmental conditions, as bivoltine silkworm breeds originated from temperate regions. Many important qualitative characters such as viability and cocoon traits decline sharply when temperature exceeds 28° C. As India has fluctuating temperature and humidity conditions, estimation of phenotypic stability at high temperature is considered of prime importance for sustainable progress in bivoltine breeding.

Previous studies have demonstrated fundamental thermotolerance in silkworms (Ueda and Lizuka 1962; Pillai and Krishnaswami, 1980, 1987; Kato et al. 1989) but information regarding the implications of these aspects of selection of parental resources for breeding programs is lacking. Some earlier studies addressed selection of silkworm breeds in respect of thermotolerance by identifying thermotolerant silkworm breeds (Shirota 1992; Tazima and Ohnuma 1995; Kumar et al. 2001). However, a clear understanding of the genetic basis and variability in the expression of quantitative and qualitative genetic traits during exposure to high temperatures is an important step for the selection of potential thermotolerance parental resources for breeding programs.

The purpose of this study is to obtain new data about screening for thermotolerance in silkworm larvae, not only to augment current knowledge on gene expression under stress conditions, but also to provide valuable information that will allow identification of thermotolerant bivoltine silkworm breeds based on the silkworm rearing performances relative to nine important economical genetic traits.

## Materials and Methods

### Silkworm breeds

The 24 bivoltine silkworm breeds used were, BD_2_, BO_1_N, BD_3_N, SOF-BR, BD_2_-S, BD_1_C-BR, BO_3_-W, SOW, SOCB, SB-F, BD_2_-G, BO_3_BL, BO_1_BL, BO_2_, SOHW, BO1BR, BD1LC, BD1O, BD_2_-LC, BD_3_, BO1-S, BD_4_, BO_3_, NB_4_D_2_. These strains with varied phenotypic and quantitative traits, including hibernation, are maintained at the Biology Division, Indian Institute of Chemical Technology, Hyderabad, AP, India.

### Silkworm rearing and estimation of genetic traits

Disease free eggs from each strain were reared and cocoons were harvested and maintained until eclosion of moths. Healthy female moths emerging on the peak day of eclosion were allowed to mate for three to four hours and held until oviposition. The eggs were acidtreated within 20 hours after oviposition, following the method developed by Yokoyama (1962) to prevent hibernation. The eggs were incubated at 25 ± 1° C temperature and 70 to 80% RH after surface treatment with 2% formalin solution. Twenty to 30 eggs were chosen from each brood and pasted onto egg sheets. Three such egg sheets for each breed were prepared, wrapped in white tissue paper and boxed with black paper to synchronize the embryonic development. On the day of hatching, the eggs were exposed to light in order to obtain uniform hatching and freshly chopped mulberry leaves were fed to the young larvae. The whole process, from silkworm egg incubation to completion of rearing activities, was carried out under hygienic conditions in a silkworm rearing laboratory thoroughly disinfected with bleaching powder and formalin solution.

Silkworm rearing was conducted for each breed in plastic boxes by feeding them the V_1_ variety of mulberry leaves from the well-maintained irrigated mulberry garden on campus. A standard rearing procedure was adopted as recommended by Datta (1992). The young larvae (l^st^–3^rd^ instars) were reared at 26–28° C with 80–90% RH and late age larvae (4^th^ and 5^th^ instars) were maintained at 24–26° C with 70–80% RH until the 3rd day of fifth instar. Each group was divided into two, one of which was maintained as a control under standard rearing conditions and the second was exposed to high temperature treatment.

### High temperature treatment

The study was carried out between March 2007 and January 2008. Silkworm rearing was conducted following the standard method under the recommended temperature and relative humidity until the second day of the fifth instar. On the third day of the fifth instar, 300 larvae per breed in three replications of 100 larvae were selected for the high temperature treatment. High temperature treatment was obtained by utilizing the NK System Biotron (www.nihonika.co.jp), an environmental growth chamber with precise and automatic control facilities for uniform maintenance of temperature and humidity. The temperature used was 36 ± 1°C and RH 50 ± 5%. Fresh mulberry leaves were given twice a day and silkworm rearing was continued as suggested by Kato et al. (1989) and Kumar et al. (2001) using appropriate plastic boxes/trays. A control group was maintained at ambient temperature of standard rearing conditions at 25 ± 1°C and RH 65 ± 5%. Thermal exposure was given every day for six hours until spinning (10:00–16:00) since continuous exposure to high temperature conditions reduces quantitative traits drastically in high thermotolerance screening experiments. Observations were carried out daily and mortality due to high temperature in each of the 24 breeds was noted. After thermal treatment, the treated silkworm larvae were shifted to the mountage for spinning at normal ambient temperature of 25 ± 2° C and RH 65 ± 5%. Cocoons were harvested 4–5 days later after completion of cocoon spinning. Harvested cocoons were accessed for survival to pupation using the equations detailed below. The pupation or survival rate was utilized as the measure of index for assessing thermotolerance by calculating the number of healthy live pupae obtained relative to the number of larvae at the beginning of the treatment both in treated and control groups.

During the process of silkworm rearing, data on larvae and cocoons for the nine genetic traits (larval weight, cocoon yield for 10,000 larvae by number and weight, pupation rate, cocoon and shell weight, shell ratio, filament length and denier) were collected and calculated according to the equations below:

**Larval weight (g)**. Mean larval weight (g) recoded for 10 randomly selected larvae at the peak of growth of fifth instar larvae from each replication. This was an indicator of the general health of the larvae.

**Cocoon yield for 10,000 larvae.** The mean number of cocoons harvested relative to the number of larvae at the beginning of the experiment, converted to 10,000 larvae.

**Cocoon yield by weight (kg) for 10,000 larvae.** The mean weight of the cocoons harvested in kilogram (kg) for every 10,000 larvae by weight.

**Pupation rate (%).** The live pupa present inside the cocoon during metamorphosis of larva into pupa expressed as a percentage. This genetic trait was kept as the ultimate index for assessing thermotolerance in this investigation.

# of good cocoons + (# of double cocoons × 2) / # of larvae beginning the experiment × 100

**Cocoon weight (g).** The average single cocoon weight in grams for 10 male and 10 female cocoons chosen randomly on the 6^th^ or 7^th^ day of spinning.

**Shell weight (g).** The average single cocoon shell weight in grams of 10 male and 10 female cocoons shell chosen randomly. The shells used were the same cocoons used for the cocoon weight determination.

**Shell ratio (%).** The total quantity of silk available from a single cocoon was expressed as a percentage using the following equation.

(single cocoon shell weight (g) / single cocoon weight (g)) × 100

**Filament length (m).** This is the most important parameter used by the industry. Silk filament length indicates the reelable length of silk filament from a cocoon. It was calculated using the average length of unwound silk filament from 10 cocoons (obtained using a mono cocoon reeling unit) and expressed in meters according to the following formula:

**Table 1.**
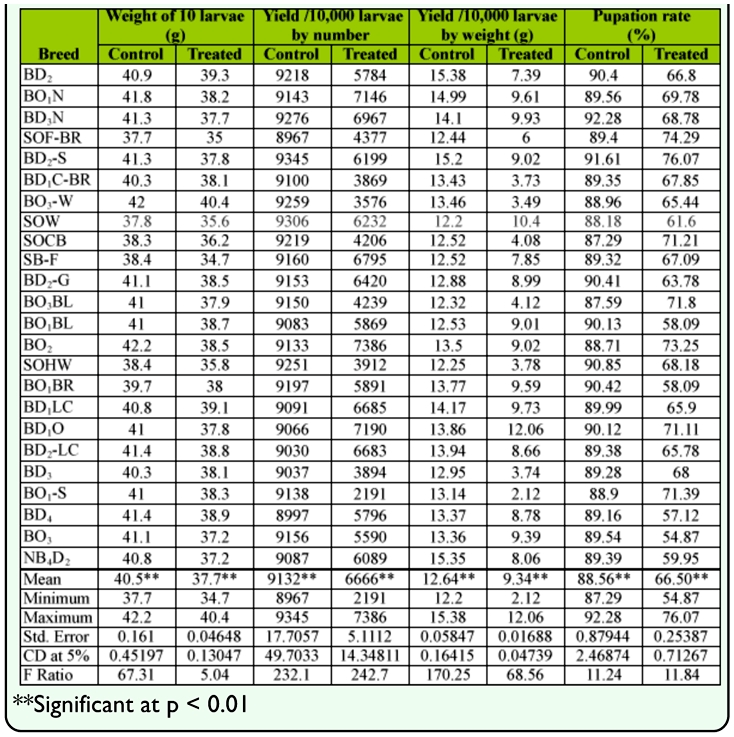
Silkworm rearing performance of bivoltine germplasm resources under normal and high temperature conditions.

length of raw silk (m) × 1.125 (circumference) / # of reeling cocoons

**Denier (d).** The thickness of the silk filament measured by following formula.

(weight of total silk filament (g) / total filament length of silk) × 9000

The data on rearing performance and post cocoon parameters of both high temperatures treated and control groups were recorded on nine genetic traits for each replicate. This was subjected to statistical analysis with assistance of ‘Indostat’ software for understanding the significance of the study by ANOVA.

## Results

### Morphological salient features of bivoltine breeds

Morphological differences were found between the germplasm breeds with respect to origin, egg, larval and cocoon traits. The 24 breeds were not of Indian origin. The chorion of 13 breeds was brown, whereas 11 breeds were pigmented, and the color of the serosa of all breeds was white. Larval marking of all breeds was plain, 19 breeds had a stout body and 5 had a slender body. Cocoon shape in 13 breeds was oval, 6 were dumbbell-shaped and 5 had peanut-shaped cocoons. Most of the breeds spun a white cocoon but SOCB and SOHW spun dull white color cocoons. Fine cocoon grains occurred in 17 breeds, medium grains occurred in 4 breeds, and coarse grains occurred in 3 breeds.

**Table 2.**
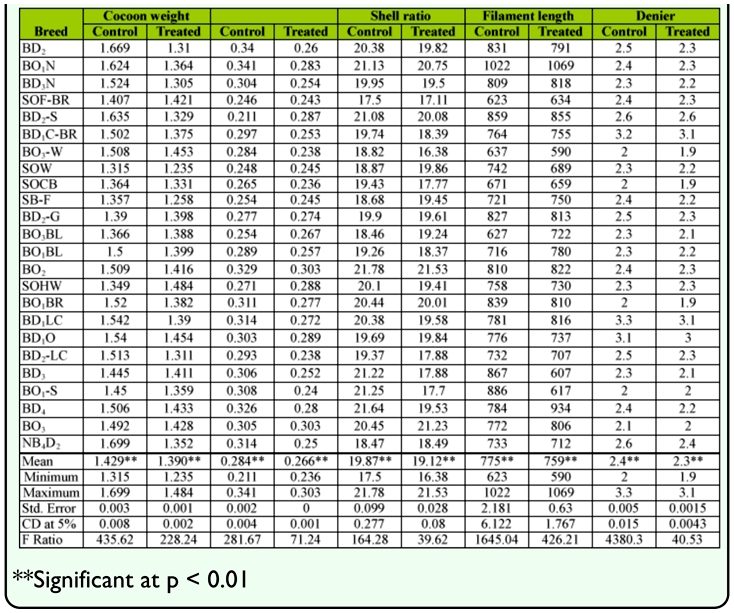
Post cocoon parameters of bivoltine germplasm resources under normal and high temperature conditions.

### Rearing performance

Considerable variations were found for 9 genetic traits among the bivoltine breeds at high temperature. Data were obtained for larva weight, yield by 10,000 larvae by number and weight, pupation, cocoon weight, shell weight, shell ratio, filament length and denier for 24 bivoltine breeds under normal and high temperature treatments. There was evidence of clear declines in all nine genetic traits in all of the high temperature treated groups (Tables 1 and 2). Among high temperature treated groups, maximum larval weight in BO_3_-W (40.4 g) followed by BD_2_ (39.3 g), BD1LC (39.1 g) and minimum in SB-F (34.7 g) with an average of 37.7 g was estimated. Highest yield /10,000 larvae by number was observed in BO_2_ (7386) and lowest in BO_1_- S (2191) with an average of 5541. Maximum yield /10,000 larvae by weight were in SOW (10.40 kg), minimum in BO_1_-S (2.12 kg) with the mean value of 7.35 kg. The highest pupation rate of 76.07% in BD_2_-S was followed by 74.29% in SOF-BR, 73.25% in BD_2_ and lowest of 54.87% in BO_3_ with a mean value of 66.51% (Table 1).

### Pupation / survival rate (index of thermotolerance)

**Figure 1.**
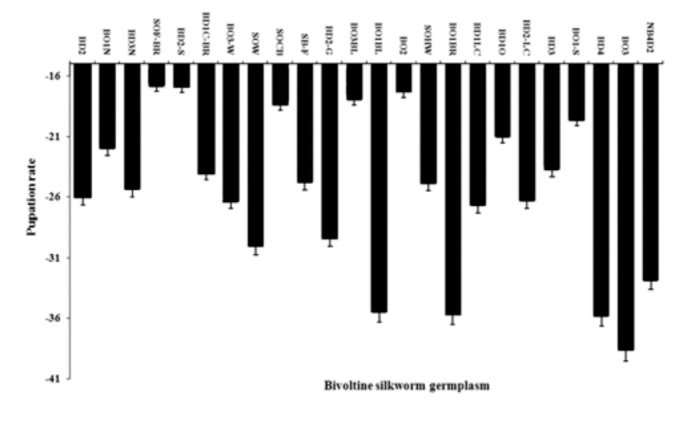
Pupation rate difference in high temperature treated groups. High quality figures are available online.

Based on the pupation/survival rate, all of the breeds were grouped for relative temperature tolerance. Among them, 7 breeds had pupation rate > 70% for the high temperature treated groups and were considered to be tolerant to high temperature (Table 1). Amongst them, maximum pupation was observed in BD_2_-S (76.07%) followed by SOF-BR (74.29%), BO_2_ (73.25%), BO_3_-BL (71.80 %), BO_1_-S (71.39%), SOCB (71.21%), BDiO (71.11%). Twelve germplasm breeds were found to exhibit < 60% of a pupation rate ranging from 69.78 % (BO_1_N) to 61.60 % (SOW) and were considered to be moderately tolerant to high temperature. While 5 germplasm breeds *viz.,* BO_1_BR, BO_1_-BL, BD_4_, BO_3_ and NB_4_D_2_ were identified as less tolerant (< 60% of pupation) to high temperature with pupation rates ranging from 58.09% (BO_1_BR) to 54.87% (BO_3_). By considering the pupation/survival rate as the index for thermotolerance, the bivoltine germplasm resource was classified as susceptible (BO_1_BR, BO_1_-BL, BD_4_, BO_3_ and NB_4_D_2_), moderately tolerant (BD_2_, BO_1_N, BD_3_N, BD_1_C-BR, BO_3_-W, SOW, SB-F, BD_2_- G, SOHW, BD_1_LC, BD_2_-LC, BD3) and tolerant (BO_1_-S, BD_2_-S, SOCB, BO_3_BL, BO_2_, BD_1_O, SOF-BR) to thermal stress using the following ranges: susceptible, 50–60%; moderately tolerant, 61–70%; and tolerant,, 71–90%. With respect to percentage difference between the high temperature groups and control, negative correlations were seen for all genetic traits (Figure 1).

### Performance of cocoon traits

Maximum single cocoon weight in SOHW was 1.484 g and minimum single cocoon weight in SOW was 1.235 g, with an average cocoon weight of 1.374 g. With respect to shell weight, the highest was 0.303 g in BO_2_ and the lowest was 0.236 g in SOCB, with an average of 0.266 g. An average of 19.14% shell ratio with the maximum of 21.53% and minimum of 16.38% was found. BO_1_N showed highest filament length of 1069 m, followed by BD_4_ with 934 m, BD_2_-S with 855 m and lowest of 590 m in BO_3_-W. The average among the breeds was 759 m. The relevant to negative correlated trait of denier reached a maximum of 3.1d in BD_1_C-BR, BD_1_LC and a minimum of 1.9d in BO_2_-W, SOCB, BO_1_BR with an average of 2.3d among bivoltine breeds (Table 2).

### **Percentage of difference in treated over control groups**

The percentage of difference among treated and control groups was the highest in BO_3_-W (-3.81%) and the lowest in SB-F (-9.64%) for the trait of larval weight. BO_2_ (19.13%) and BD_1_-S (76.02%) showed high and low percentages of difference for the traits of cocoon yield by number, respectively. Maximum difference occurred in BD_1_O (12.99%) and minimum difference in BO_1_-S (83.87%) for cocoon yield by weight. With respect to cocoon weight, a positive difference in SOHW (10.01%) and negative difference in BD_2_ (-21.51%) was found. For shell weight, a maximum positive difference was found in BD_2_-S (36.02%) and a minimum positive difference was found in BD_2_ (-23.53%). For shell ratio, a high positive difference was found in SOW (5.25%) and a low positive difference was found in BO_1_-S (-16.72%). With respect to filament length, maximum positive difference occurred in BD_4_ (19.13%) and minimum positive difference occurred in BO_1_-S (-30.36%). For the trait of denier, a high difference occurred in BO_1_-S (-0.76%) and low difference occurred in BD_4_ (-11.29%) (Table 3).

**Table 3.**
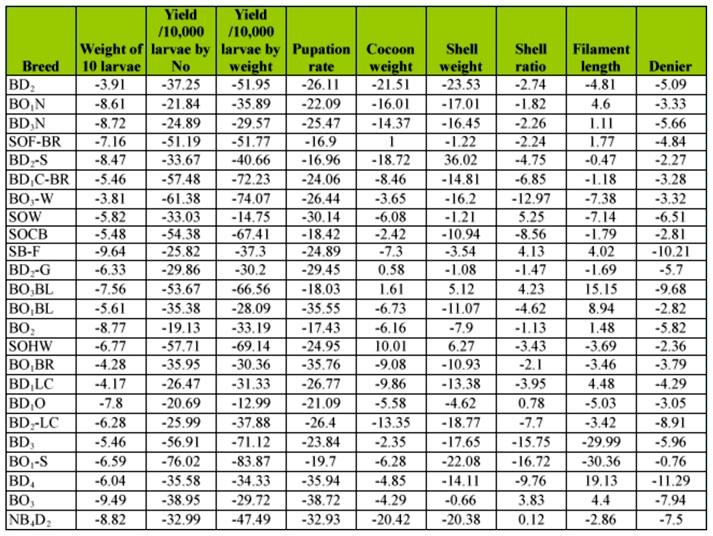
Percentage of difference on genetic traits in bivoltine germplasm resources of high temperature conditions groups over control.

### Positive percentage difference on genetic traits

**Figure 2.**
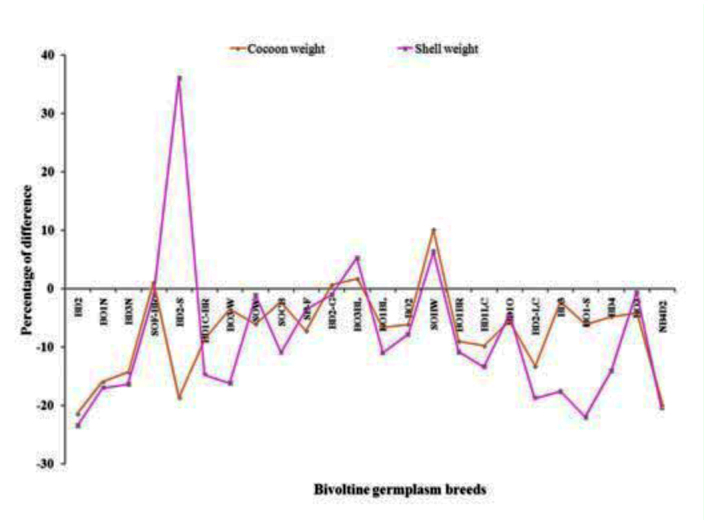
Cocoon and shell weight difference in high temperature treated groups over control. High quality figures are available online.

**Figure 3.**
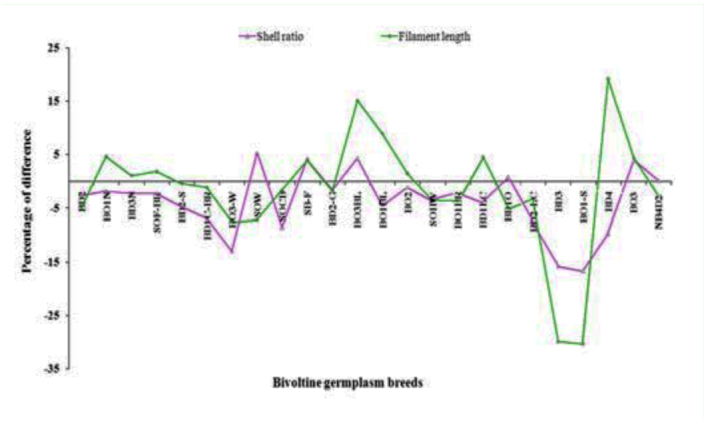
Shell ratio and filament length difference in high temperature treated groups and control. High quality figures are available online.

**Figure 4.**
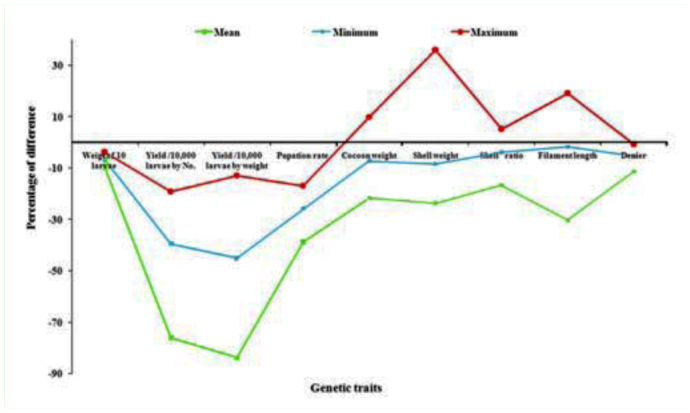
Percentage difference among genetic traits in high temperature treated groups over control. High quality figures are available online.

Among the 9 genetic traits of breeds, 4 important traits such as cocoon weight, shell weight, shell ratio and filament length were found to exhibit the positive difference in the high temperature treated group over the control group (Table 3). In the case of cocoon weight, positive percentage differences were observed in SOHW (10.01%), followed by BO_3_ BL (1.61%), SOF-BR (1.00%), BD_2_-G (0.58%) (Figure 2). The maximum difference in BD_2_-S (36.02%), which was followed by SOHW (6.27%) and BO_3_BL (5.12%) was revealed on shell weight (Figure 3). High positive difference in SOW (5.25%) followed by BO_3_BL (4.23%), SB-F (4.13%), BO_3_ (3.83%) and BD_1_O (0.78%), NB_4_D_2_ (0.12%) occurred on shell ratio (Figure 3). For filament length, maximum difference occurred in BD_4_ (19.13%) followed by BO_3_BL (15.15%), BO_1_BL (8.94%), BO_1_N (4.60%), BDiLC (4.48%), BO_3_ (4.40%), SB-F (4.02%), SOF-BR (1.77%), BO_2_ (1.48%) and BD_3_N (1.11%) (Figures 2 and 4).

The collected data for the 9 genetic traits were statistically analyzed by the variance test for significant differences between the normal and treated groups. Results obtained after analysis of variance of mean squares revealed highly significant (p < 0.001) differences among all genetic traits for the breeds. The maximum critical difference of 5% was demonstrated for the trait of yield/10,000 larvae by number (14.3481) among high temperature treated groups, which was followed by filament length (1.767), pupation rate (0.71267) yield /10,000 larvae by weight, filament length (6.122) and shell weight (0.001) (Tables 1 and 2). All genetic traits showed a decline with increase of temperature compared to the controls.

Based on all morphological genetic traits along with the maximum thermotolerance level on pupation rate, three bivoltine silkworm germplasm breeds *viz.,* BD_2_-S, SOF-BR and BO_2_ ,were identified as potential thermo tolerant breeding resources for breeding programs.

## Discussion

On thermal treatment, all genetic traits of silkworms showed a decline with the increase of temperature above standard level. A similar result was reported by Kumar et al. (2002). They found that biological molecules like DNA, RNA, lipids, etc. were vulnerable to heat stress. Temperature stress causes a number of abnormalities at the cellular level as the normal pattern of protein synthesis halts. Another important effect of temperature (or stress of any kind) is the unfolding of cellular proteins. Cellular proteins are typically folded in their native conformations while functioning in cells. This process can result in aggregates of unfolded protein that at best diminish the pool of functional proteins and at worst are cytotoxic (Feder 1996; Feder et al. 1996). However, a brief exposure of cells to sub-lethal high temperature was found to render protection to the organism of subsequent and more severe temperature changes (Denlinger and Yoccum 1998; Gilchrist and Huey 1999; Kevin 2002). In a study with heat-shocked *Drosophila,* continued survival and acclimatization of the experimental insects occurred at higher temperatures (Dingley and Maynard Smith 1968). Further, every form of stress is known to induce a set of proteins in all tested organisms from bacteria to man. Inducing stressors include ethanol, heavy metals, hypoxia, hyperoxia, changes in pH, free radicals, various poisons and toxins, ischemia, osmotic shock, ionizing radiation and many others (Feder 1996). The heat shock response in *B. mori* cells induced active transcription of heat shock mRNAs (Evegnev et al. 1987; Kiuchi et al. 2007). It was reported that 93, 70, 46 and 28 kDa protein bands appeared after exposure to high temperature in both bivoltine and polyvoltine strains, but with varying kinetics. The isolated hemocytes of polyvoltine breeds exhibited the induction of 70 kDa protein (Joy and Gopinathan 1995). For instance, polyvoltine breeds reared in tropical countries are known to tolerate slightly higher temperature, as are cross breeds that have evolved for a tropical climate (Ramesha et al. 2009).

The success of the sericulture industry depends upon several variables, but environmental conditions such as biotic and abiotic factors are of particular importance. Among the abiotic factors, temperature plays a major role on growth and productivity of silkworms (Benchamin and Jolly 1986). It is also known that the older silkworms prefer relatively lower temperature than younger silkworms and that fluctuation of temperature during different stages of larval development was found to be more favorable for growth and development of larvae than constant temperature. There is ample literature stating that good quality cocoons are produced within a temperature range of 22-27° C and that cocoon quality is poorer above these levels (Krishnaswami et al. 1973; Datta 1992; Datta et al. 1996, 1997). However, polyvoltine breeds reared in tropical countries are known to tolerate slightly higher temperature (Hsieh et al. 1995), as are cross breeds that have been developed for tropical climates. In order to use bivoltine races in a tropical country like India, it is necessary to have a stable cocoon crop in a high temperature environment. High temperature affects nearly all biological processes including the rates of biochemical and physiological reactions (Hsieh et al. 1995; Willmer et al. 2004), and can eventually affect the quality or quantity of cocoon crops in the silkworm. Several reports (Ueda and Lizuka 1962; Shirota 1992; Tazima and Ohuma 1995; Hsieh et al. 1995) demonstrated that silkworms were more sensitive to high temperature during the fourth and fifth stages, which are recommended for the recognition and selection of thermo tolerant silkworm breeds, under high temperature conditions. Recently, the effects of temperature on the development and survival of the Argentine ant also have been shown by Abril et al. (2010).

It is well understood that the majority of the economically important genetic traits of silkworms are qualitative in nature and that phenotypic expression is greatly influenced by environmental factors such as temperature, relative humidity, light and nutrition (Krishnaswami et al. 1971; Pillai and Krishnaswami 1980, 1987; Wu and Haul993; Tazima 1984; Thaigarajan et al. 1993; Zhang et al. 2002; Zhao et al. 2007; Ramesha et al. 2010). Therefore, it is essential to gauge the degree of phenotypic difference of the economical traits to understand the genetic steadiness under the varied environmental conditions and the productivity of germplasm breeding resources. The problem of balancing and fixing the desirable traits for a given environment is a challenging task for the breeder. Hence, understanding the range of reactions of selected breeds to variable environmental conditions is important for the breeder to utilize them appropriately in hybrid programs. Intensive and careful domestication over centuries has apparently deprived the insect of opportunities to acquire thermotolerance. Among many factors responsible for poor performance of the bivoltine strains under tropical conditions, the main culprit is temperature. Indeed, many quantitative characters decline sharply when temperature is higher than 28° C. Breeders in the field agree that it is a difficult task to breed such bivoltine breeds that are suitable to high temperature environments with productive traits. It is a well-established fact that under tropical conditions, bivoltine races are more vulnerable to various stresses including climatic conditions, poor leaf quality and improper management during the summer. In order to efficiently select breeds with high temperature tolerance, it is important to analyze the impact of high temperature on many genetic traits of these silkworm breeds.

In order to achieve greater success in this regard, it is important to understand the level of temperature tolerance in silkworm bivoltine breeds. The main objective of this study was to identify bivoltine silkworm breeds tolerant to high temperature among 24 germplasm resources evaluated for tolerance to thermal stress at 36 ± 1°C and relative humidity (RH) 50 ± 5% analysis by measuring qualitative and quantitative genetic traits for three successive generations. The results obtained support the earlier observation of Kumar et al. (1989), Kato et al. (1989), Koundinya et al. (2003). The latter authors' emphasis was on the phenotypic manifestations of 9 genetic traits under high temperature conditions. The results revealed highly significant (p < 0.01) variability among the germplasm resources of bivoltine silkworm germplasm with respect to nine genetic traits on treated groups relating to survival or pupation rate. The benchmark for short listing toward thermotolerance was kept as pupation rate of > 70% for bivoltine as suggested by Kumar et al. (2002). Although earlier studies have shown that breeds like Hosa Mysore, KA and their hybrids are moderately tolerant to high temperature up to 38°C (Pillai and Krishnaswami 1987; Shirota 1992), the current study indicates that seven bivoltine germplasm were identified as temperature tolerant with a pupation rate of above 70%.

This study shows less percentage decline in BO_3_W, BO_2_, BD_1_O, BD_1_O for larval weight, cocoon yield by number, by weight, and pupation rate respectively, better performance over control in BD_2_-G, SOF-BR, BO_3_BL, BD_2_-S, SOHW for shell weight, and in NB_4_D_2_, BD_1_O, SB F, BO_3_-BL for shell ratio with maximum pupation rates in BD_2_-S, SOFBR, BO_2_, BO_3_-BL, BD_1_O indicating a capacity for thermotolerance.

In view of the above observations, it was a difficult task to break the negative correlation associated with survival rate and productivity traits. In the present study, bivoltine germplasm resources with maximum pupation or survival rate were identified as potential breeding resource material for the development of thermotolerant breeds/hybrids. This resulted in the identification of BD_2_-S, SOF-BR, and BO_2_ as temperature tolerant bivoltine germplasm resources based on their pupation or survival rate as the index and their performance on nine quantitative genetic traits. These resources are recommended as potential breeding resources for the development of thermotolerant silkworm breeds/ hybrids.
